# Comparison of Haploidentical Hematopoietic Stem Cell Transplant With or Without Unrelated Cord Blood Infusion in Severe Aplastic Anemia: Outcomes of a Multicenter Study

**DOI:** 10.3389/fimmu.2022.912917

**Published:** 2022-06-23

**Authors:** Meiqing Lei, Yanming Zhang, Wenjing Jiao, Xiaoli Li, Huifen Zhou, Qingyuan Wang, Huiying Qiu, Xiaowen Tang, Yue Han, Chengcheng Fu, Zhengming Jin, Suning Chen, Aining Sun, Miao Miao, Limin Liu, Depei Wu

**Affiliations:** ^1^The First Affiliated Hospital of Soochow University, Jiangsu Institute of Hematology, Key Laboratory of Thrombosis and Hemostasis of Ministry of Health, Collaborative Innovation Center of Hematology, Suzhou, China; ^2^Department of Hematology, Haikou Municipal People’s Hospital, Affiliated Haikou Hospital of Xiangya Medical College, Central South University, Haikou, China; ^3^Department of Hematology, The Affiliated Huai’an Hospital of Xuzhou Medical University and the Second People’s Hospital of Huai’an, Huai’an, China; ^4^Department of Hematology, Xian Yang Central Hospital, Xianyang, China; ^5^Soochow Hopes Hematonosis Hospital, Suzhou, China

**Keywords:** Severe aplastic anemia, Haploidentical donor, hematopoietic stem cell transplant, unrelated cord blood, Comparision

## Abstract

The purpose of this study in severe aplastic anemia (SAA) patients was to compare the feasibility and efficacy of haploidentical hematological stem cell transplantation combined with a single unrelated cord blood (UCB) infusion (Haplo-cord-HSCT) or haplo-identical HSCT (Haplo-HSCT) alone. The five-year graft-versus-host disease (GVHD)-free or failure-free survival (GFFS) was similar between the two groups (72.4 ± 3.4% vs. 65.4 ± 5.2%, P = 0.178); however, the five-year overall survival (OS) was more favorable in the Haplo-cord-HSCT group than that in the Haplo-HSCT group (84.0 ± 2.8% vs. 72.6 ± 4.9%, P = 0.022), as was transplantation-related mortality (16.4% vs. 27.4%, P = 0.039). Multivariate analysis showed that Haplo-cord HSCT was the only independent determinant of increased OS (P = 0.013). Explorative subgroup analysis showed that only an Human leukocyte antigen-A (HLA-A) allele match between UCB and the recipient was a beneficial factor for GFFS in the Haplo-cord-HSCT group (P = 0.011). In the haplo-cord with an HLA-A match (n = 139) or mismatch (n = 32) or Haplo-HSCT groups, a haplo-cord HLA-A allele match was associated with lower I–IV and III–IV acute GVHD. The haplo-cord with an HLA-A match subgroup also had higher five-year OS than the Haplo-HSCT group (85.4 ± 3.0% vs. 72.6 ± 4.9%, P = 0.013), and higher five-year GFFS than the Haplo-cord HLA-A allele mismatch subgroup (76.2 ± 3.6% vs. 56.3 ± 8.8%, P = 0.011). These findings suggest that the coinfusion of a single UCB potentially improves survival of Haplo-HSCT in SAA patients and that an HLA-A allele-matched UCB is the preferred option.

## Introduction

Allogeneic hematological stem cell transplantation (allo-HSCT) from an available matched sibling donor (MSD) is recommended as a first-line treatment for patients with acquired severe aplastic anemia (SAA), particularly in young patients (1). Unfortunately, less than 30% of patients who require an allo-HSCT have a human leukocyte antigen (HLA)-compatible sibling. Fortunately, almost everyone can be matched to at least one related HLA-haploidentical donor (HID). With recent advances in transplant technology, haploidentical HSCT (haplo-HSCT) has become an important alternative treatment for SAA patients who do not have a suitable identical donor and for those who are refractory to immunosuppressive therapy (IST) (2, 3). The overall survival (OS) rate for haplo-HSCT ranges from 67.1% to 89.0% in SAA patients, with this high value comparable to that for MSD transplantation (4–6). Nevertheless, the outcome of a haplo-HSCT in SAA patients is still limited by transplantation-related mortality (TRM) caused by graft failure (GF) or graft-versus-host disease (GVHD) and infections associated with delayed immune reconstitution (7, 8). If a way to reduce the risk of these complications was discovered, the efficacy of haplo-HSCT would be further improved.

In recent years, some experienced transplant centers have been investigating strategies to optimize the haplo-HSCT model, such as haplo-HSCT combined with mesenchymal stem cells (MSCs), umbilical cord blood (UCB), or a post-transplant cyclophosphamide conditioning regimen (9–11). It should be noted that the conditioning regimen, the time and count of UCB infusion, and the mechanism of allo-HSCT success in different hematological diseases are sometimes different (12–15). Until recently, our two studies not only demonstrated that a haplo-cord-HSCT achieved 97.1% donor myeloid engraftment and 81.4 ± 3.3% four-year OS in SAA patients but also that failure-free survival and a health-related quality of life were both better in SAA patients after first-line haplo-cord-HSCT than that achieved by IST (16, 17). However, to the best of our knowledge, there has been no direct comparison of the therapeutic outcomes of haplo-HSCT with or without a UCB infusion in SAA patients. We therefore carried out a multicenter cohort study to retrospectively compare these two treatment modalities in this specific disease.

## Subjects and Methods

### Patients

Between March 2011 and August 2020, 255 consecutive acquired SAA or very SAA (vSAA) patients were enrolled in this study from five transplant centers. All patients met the following diagnosis and management guidelines for SAA or vSAA (1, 18) (1): no MSD or matched unrelated donor (MUD), or not willing to wait for an MUD (2); no response to previous IST [including anti-thymocyte globulin (ATG)/antilymphocyte immunoglobulin (ALG) plus cyclosporine A] (3); having one or more available HIDs, and willing to choose haplo-HSCT as the first-line or alternative treatment (4); transfusion dependent (5); a Karnofsky score of 80−100; and (6) the absence of severe liver, renal, lung, and heart diseases. The patients were classified into two groups based on whether they have received a single UCB infusion before the haploidentical grafts (haplo-cord-HSCT, n = 171 and haplo-HSCT, n = 84). Every patient signed a written, informed consent form prior to participation. The study was approved by our hospital’s Ethics Committee and was carried out in accordance with the Declaration of Helsinki.

### HLA Typing and Donor Selection

The HLA-A, -B, -C, -DRB1, and -DQB1 typing of recipients and donors including their parents, siblings, or children were matched at the allele level using a high-resolution molecular standard technique. HIDs were selected based on matched HLA with a true haploid genetic background, age, gender, health condition, and willingness to donate stem cells. In addition, the donors were excluded if the recipient had donor-specific antibodies (DSAs) against high-expression HLA, with a mean fluorescence intensity >2,000. If a targeted positive donor was the only choice, rituximab and/or plasma exchange were administered to the recipient prior to transplantation.

The HLA-A, -B, and -DRB1 typing of UCB units obtained from the cord blood bank in Shanghai, China was performed. The choice of UCB was according to the treating physician’s discretion, depending on the availability of a suitable UBC and the patient’s preference. As in our previous report (17), UCB units with two or less mismatchings in the HLA-A, -B, and -DRB1 loci were selected as minimum candidates. Priority was given to units with the most closely matched HLA and subsequently to the unit with the highest cell count with a similar degree of matching for the HLA type. The HLA typing and cell count of the selected UCB units were reassessed after rapid thawing at our centers. If no suitable UCB was found according to the above principles, the recipient was placed in the haplo-HSCT group.

### Conditioning Regimen

The transplant schedule was described as follows: the first day of the stem cell infusion was designated as “day 01” and the second day of infusion as “day 02.” The specific days before the first and last stem cell infusion were designated by a minus (–) sign and plus (+) sign, respectively. All patients were treated with a busulfan (BU)/cyclophosphamide (CY)-based regimen as described in our previous report (17): busulfan (Bu, intravenous, total dose 6.4 mg/kg, days –7, –6), cyclophosphamide (Cy, intravenous, total dose 200 mg/kg, days –5 to –2), and rabbit ATG (rATG, intravenous, total dose 10.0 mg/kg, days –5 to –2) or porcine antihuman lymphocyte immunoglobulin (pALG, intravenous, total dose 80.0 mg/kg, days –5 to –2).

### Graft Collection and Infusion

From day –4 to the last day of stem cell collection, hematopoietic stem cells from the HIDs were mobilized using the recombinant human granulocyte colony-stimulating factor (rhG-CSF) at a dose of 10 μg/kg/day. BM grafts were collected *via* BM aspiration in the surgery room on day 01, with the target count for mononuclear cells (MNCs) set at 2–4 × 10^8^/kg of the recipient’s weight. Peripheral blood (PB) grafts were collected by apheresis using a COBE Spectra device (Gambro BCT, Lakewood, CO, USA) on day 02. The total MNC count from BM and PB were required to achieve 6–8 × 10^8^/kg of the recipient’s weight. If the count of total MNCs was still not at this level, more PBSCs were collected on the next 1–2 days. Unmanipulated grafts from BM and PB were infused into the recipient on the day of collection. In the haplo-cord HSCT group, a single UCB infusion was conducted 8 h prior to haploidentical graft infusion on day 01.

### GVHD Prophylaxis and Treatment

Cyclosporine A, mycophenolate mofetil (MMF), and short-term methotrexate (MTX) were administered for acute GVHD (aGVHD) prophylaxis in the two groups. Once aGVHD or chronic GVHD (cGVHD) occurred, the corresponding treatment was given as described in our previous report (17).

### Supportive Care and Post-Transplantation Surveillance

The details of supportive care and post-transplantation surveillance were in line with our previous experience (17).

### Definitions and Post-Transplantation Evaluations

Neutrophil engraftment was defined as the first day of an absolute neutrophil count (ANC) >0.5 × 10^9^/L on three consecutive days. Platelet engraftment was defined as the first day of a platelet count >20 × 10^9^/L during a week without platelet transfusion. Primary GF was defined as the failure to achieve neutrophil engraftment after HSCT up to day +28, while secondary GF was defined as an ANC <0.5 × 10^9^/L on three consecutive time points after the confirmation of initial complete donor engraftment (19). Delayed platelet recovery was defined as platelet engraftment achieved after more than +30 days. The diagnosis and severity of aGVHD and cGVHD were based on established criteria (20, 21). On the premise of full donor chimerism without relapse or severe GVHD, poor graft function was defined as persistent cytopenia in at least two lineages (platelet <20 × 10^9^/L, neutrophil count <0.5 × 10^9^/L, hemoglobin level <70 g/L) and/or requiring a transfusion beyond +28 days (22). TRM was defined as death related to the transplantation instead of SAA relapse. GVHD-free or failure-free survival (GFFS) was defined as survival without grade III–IV aGVHD, moderate-to-severe cGVHD, or treatment failure including death, primary or secondary GF, and relapse. After transplantation, the recipients’ BM was reexamined monthly for 3 months and every 3–6 months for the following 1–2 years.

### Statistical Analysis

The date of the last follow-up for all surviving patients was June 30, 2021. SPSS 22.0 statistical software (IBM, Armonk, NY, USA) was used for the statistical analyses. Continuous and categorical variables of demographic-, disease-, and treatment-related factors were compared using the Mann–Whitney U and Pearson chi-squared tests, respectively. GVHD was estimated as a cumulative incidence, considering early death and GF as competing events. Survival analysis was conducted using the Kaplan–Meier method and log-rank test. Factors with a *P*-value <0.05 in the univariate analysis were included in a Cox regression multivariate analysis. To examine the impact of a UCB HLA-loci mismatch, the variable of primary interest, a three-group comparison was carried out to construct a Cox proportional hazards model: haplo-cord with an HLA-A allele match vs. haplo-cord with an HLA-A allele mismatch vs. haplo-HSCT. *P*-values <0.05 were considered statistically significant.

## Results

### Patient Characteristics

A comparison of the clinical characteristics of the patients and donors (grafts) in the haplo-HSCT and haplo-cord-HSCT groups is shown in **Table 1**. There were no differences in recipient sex/age, disease status, PNH (paroxysmal nocturnal hemoglobinuria) clone, previous treatment, time from diagnosis to allo-HSCT, donor–recipient relationship, the blood types of donors to recipients, the source of haploidentical graft, the count of MNCs, and CD34^+^ cells from the haploidentical grafts. Notably, the donors in the haplo-HSCT group were younger than those in the haplo-cord-HSCT group (median age 36.5 vs. 41 years, *P* = 0.045). The proportion of male donors in the haplo-HSCT group was higher than that in the haplo-cord-HSCT group (75.0% vs. 62.6%; *P* = 0.048). The median count of total nucleated cells (TNCs) and CD34^+^ cells in the UCB of the haplo-cord-HSCT group was 1.80 × 10^7^/kg and 0.48× 10^5^/kg of the recipient’s weight, respectively.

**Table 1:** Patient and donor (graft) characteristics and clinical outcomes between the two groups.

Variables	Haplo-HSCT (n = 84)	Haplo-cord-HSCT (n = 171)	*P*
Median age, years (range)	25 (3–50)	25 (7–55)	0.945
Age, no. (%)			0.102
≤ 20 years	32 (38.1)	57 (33.3)	
20–40 years	35 (41.7)	93 (54.4)	
≥ 40 years	17 (20.2)	21 (12.3)	
Gender (male/female), no.	56/28	99/72	0.178
Disease status (SAA/vSAA), no.	47/37	92/79	0.746
With PNH clone, no. (%)	16 (19.0)	18 (10.5)	0.060
Previous treatment			0.613
CsA ± ATG/ALG ± others, no. (%)	19 (22.6)	34 (19.9)	
Supportive treatment, no. (%)	65 (77.4)	137 (80.1)	
Time from diagnosis to HSCT, months median (range)	4.0 (0.7–216.0)	2.0 (0.5–240.0)	0.090
Donor median age, years (range)	36.5 (11.0–63.0)	41.0 (8.0–63.0)	**0.045**
Donor-recipient sex match, no. (%)			0.118
Male-male	42 (50.0)	68 (39.8)	
Male-female	21 (25.0)	39 (22.8)	
Female-male	14 (16.7)	31 (18.1)	
Female-female	7 (8.3)	33 (19.3)	
Donor sex, no. (%)			**0.048**
Male	63 (75.0)	107 (62.6)	
Female	21 (25.0)	64 (37.4)	
Donor-recipient relationship, no. (%)			0.081
Mother-child	7 (8.3)	36 (21.1)	
Father-child	34 (40.5)	62 (36.3)	
Child-mother	9 (10.7)	10 (5.8)	
Child-father	7 (8.3)	9 (5.3)	
Siblings	27 (32.1)	54 (31.8)	
Blood types of donor to recipient, no. (%)			0.426
Matched	42 (50.0)	91 (53.2)	
Minor mismatch	21 (5.0)	36 (21.1)	
Major mismatch	13 (15.5)	35 (20.5)	
Major and minor mismatch	8 (9.5)	9 (5.3)	
Source of graft, no. (%)			0.234
BM	3 (3.6)	16 (9.4)	
PB	7 (8.3)	16 (9.4)	
BM + PB	74 (88.1)	139 (81.2)	
Median BM/PB MNCs, × 10^8^/kg (range)	11.3 (3.9–26.9)	11.4 (3.6–33.4)	0.226
Median BM/PB CD34^+^ cells, × 10^6^/kg (range)	4.4 (1.5–14.4)	3.6 (0.7–9.9)	0.342
HLA compatibility of UCB, no. (%)			–
4/6	–	46 (26.9)	–
5/6	–	91 (53.2)	–
6/6	–	34 (9.9)	–
Median UCB TNCs, × 10^7^/kg (range)	–	1.8 (0.1–6.3)	–
Median UCB CD34^+^ cells, × 10^5^/kg (range)	–	0.5 (0.1–2.3)	–
Engraftment			
Median days to ANC > 0.5 × 10^9^/L (range)	12 (9–27)	11 (9–24)	0.381
Median days to PLT > 20.0 × 10^9^/L (range)	15 (8–101)	15 (9–330)	0.828
Primary GF, no. (% of evaluable patients)	2 (2.6)	2 (1.2)	0.801
Secondary GF, no. (% of evaluable patients)	1 (1.3)	1 (0.6)	0.534
Delayed platelet recovery, no. (%)	9 (11.7)	12 (7.2)	0.250
PLT GF, no. (%)	3 (3.9)	4 (2.4)	0.816
Poor engraftment function, no. (%)	1 (1.3)	3 (1.8)	1.000
Infection			
Bacteria and fungi	54 (64.3)	101 (59.1)	0.422
CMV viremia	26 (31.0)	50 (29.2)	0.779
EBV viremia	14 (16.7)	24 (14.0)	0.579
TRM, no. (%)	23 (27.4)	28 (16.4)	**0.039**
Primary GF, no. (%)	1 (1.2)	2 (1.2)	0.988
Secondary GF, no. (%)	1 (1.2)	1 (0.6)	0.606
aGVHD, no. (%)	5 (6.0)	8 (4.7)	0.664
cGVHD, no. (%)	1 (1.2)	2 (1.2)	0.988
Infection, no. (%)	11 (13.1)	8 (4.7)	**0.016**
TA-TMA, no. (%)	1 (1.2)	2 (1.2)	0.988
Intracranial hemorrhage, no. (%)	1 (1.2)	4 (2.3)	0.534
MDS, no. (%)	0 (0.0)	1 (0.6)	1.000
PTLD, no. (%)	1 (1.2)	0 (0.0)	1.000
Other, no. (%)	1 (1.2)	0 (0.0)	1.000
Median follow-up time in survivors, months (range)	62 (11–156)	56 (12–117)	0.407

Haplo-cord HSCT, haploidentical hematopoietic stem cell transplantation with unrelated cord blood infusion; SAA, severe aplastic anemia; vSAA, very SAA; PNH, paroxysmal nocturnal hemoglobinuria; CsA, cyclosporine A; ATG, anti-thymocyte globulin; ALG, anti-lymphocyte immunoglobulin; BM, bone marrow; PB, peripheral blood; MNCs, mononuclear cells; TNCs, total nucleated cells; ANCs, absolute neutrophil count; UCB, umbilical cord blood; TRM, transplantation-related mortality; PLT, platelet; aGVHD, acute graft-versus-host disease, cGVHD, chronic GVHD; GF, graft failure; MDS, myelodysplastic syndrome; TA-TMA, transplantation-associated thrombotic microangiopathy; CMV, cytomegalovirus; EBV, Epstein– Barr virus; PTLD, post-transplant lymphoproliferative disease. The bold values means P with statistical significance.

### Engraftment

A total of 77 and 166 patients (evaluable engraftment) survived for longer than +28 days in the haplo-HSCT and the haplo-cord-HDCT groups, respectively. The median time of neutrophil engraftment in the haplo-HSCT (75/77) and the haplo-cord HSCT (164/166) was 12 (range, 9–27) and 11 (range, 10–20) days (*P* = 0.381), respectively, with all achieving complete haploidentical chimerism only, without the evidence of UCB or mixed engraftment. The primary GF rates in the haplo-HSCT (2/77) and haplo-cord-HSCT groups (2/166) were 2.6% and 1.2%, respectively (*P* = 0.801). The corresponding secondary GF rates in the haplo-HSCT (1/77) and haplo-cord-HSCT groups (1/166) were 1.3% and 0.6% (*P* = 0.534). Of the 6 patients with GF, 5 patients died of GF, while 1 patient with primary GF survived with a dependence on blood transfusion (**Table 1**). The median time to achieve platelet engraftment was 15 (range, 8–101) days in the haplo-HSCT group and 15 (range, 9–330) days in the haplo-cord-HSCT groups (*P* = 0.828). The delayed platelet recovery rates in the haplo-HSCT (9/77) and haplo-cord-HSCT groups (12/166) were 11.7% and 7.2%, respectively (*P* = 0.250). A total of 3 patients in the haplo-HSCT group and 4 in the haplo-cord-HSCT group experienced platelet GF (3.9% vs. 2.4%, *P* = 0.816), while 1 patient in the haplo-HSCT group and 3 patients in the haplo-cord-HSCT group had poor graft function (1.3% vs. 1.8%, respectively, *P* = 1.000) (**Table 1**).

### GVHD Incidence and Severity

**Figure 1:**
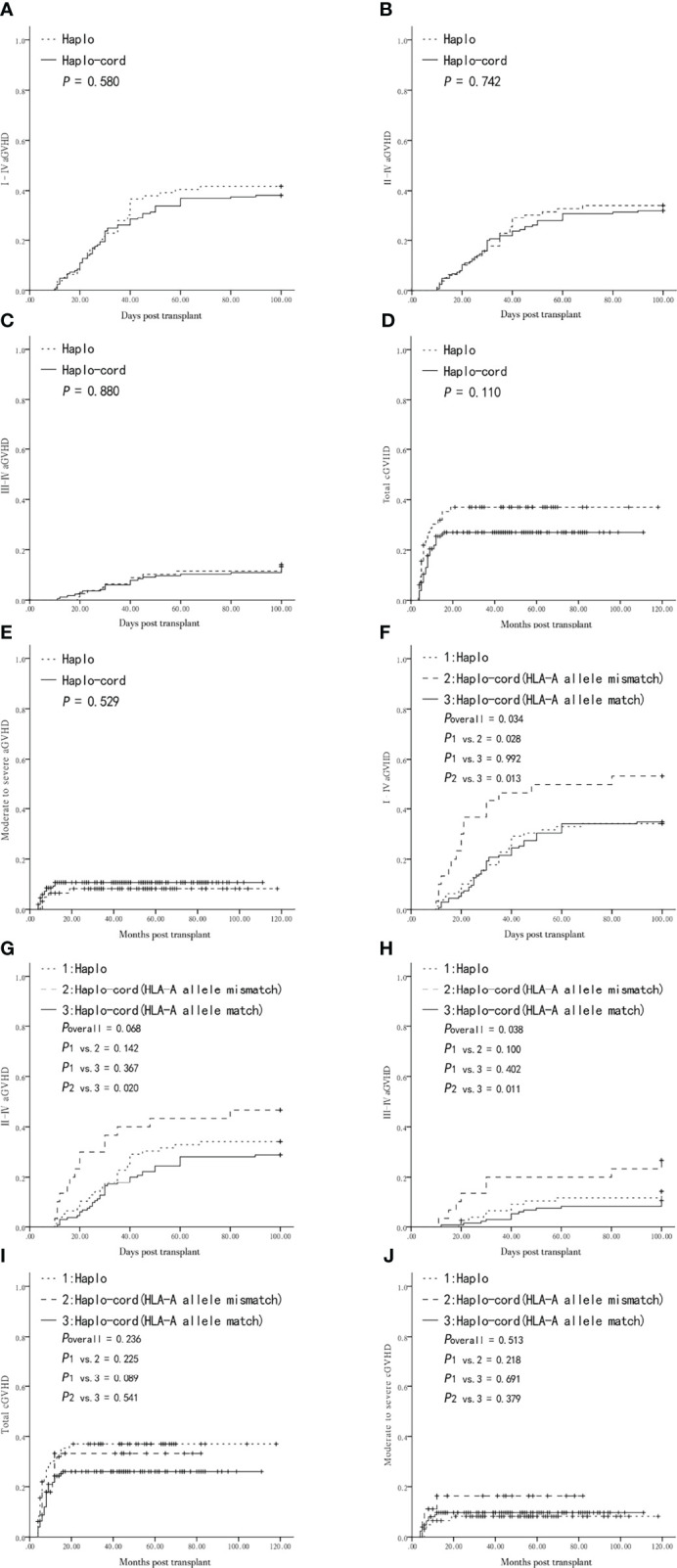
Graft-versus-host-disease (GVHD) after transplantation from Haplo-HSCT or Haplo-cord-HSCT (including subgroups). **(A)** Grade I–IV acute GVHD (aGVHD) between Haplo-HSCT and Haplo-cord-HSCT group. **(B)** Grade II–IV aGVHD between the two groups. **(C)** Grade III–IV aGVHD between the two groups. **(D)** Total chronic GVHD (cGVHD) between the two groups. **(E)** Moderate to severe cGVHD between the two groups. **(F)** Grade I–IV aGVHD among Haplo-HSCT group and Haplo-cord with a HLA-A match or mismatch subgroups. **(G) **Grade II–IV aGVHD among Haplo-HSCT group and Haplo-cord with a HLA-A match or mismatch subgroups. **(H)** Grade III–IV aGVHD among Haplo-HSCT group and Haplo-cord with a HLA-A match or mismatch subgroups. **(I)** Total cGVHD among Haplo-HSCT group and Haplo-cord with a HLA-A match or mismatch subgroups. **(J)** Moderate to severe cGVHD among Haplo-HSCT group and Haplo-cord with a HLA-A match or mismatch subgroups.

The cumulative incidence of grade I–IV, II–IV, and III–IV aGVHD was not different between the haplo-HSCT and haplo-cord-HSCT groups (41.8 ± 5.5% vs. 38.2 ± 3.8%, *P* = 0.580, **Figure 1A**; 34.2 ± 5.3% vs. 32.1 ± 3.6%, *P* = 0.742, **Figure 1B**; and 14.1 ± 3.9% vs. 13.3 ± 2.6%, *P* = 0.880, **Figure 1C**). The patients who survived for longer than +100 days were evaluated for cGVHD. The five-year cumulative incidence of overall cGVHD tended to be higher in the haplo-HSCT group compared with that in the haplo-cord-HSCT group, although this difference was not statistically significant (37.0 ± 6.2% vs. 27.0 ± 3.7%, *P* = 0.110, **Figure 1D**). The five-year cumulative incidence of moderate-to-severe cGVHD after a haplo-HSCT or haplo-cord-HSCT was also similar (8.2 ± 3.5% vs. 10.7 ± 2.5%, respectively, *P* = 0.529, **Figure 1E**).

The haplo-cord-HSCT group was divided into haplo-cord with HLA-A allele mismatched and matched subgroups, for comparison with the haplo-HSCT group. The cumulative incidence of grade I–IV aGVHD was higher in the haplo-cord with HLA-A allele mismatch subgroup than that in the haplo-cord with HLA-A allele matched or haplo-HSCT groups (53.3 ± 9.1% vs. 34.8 ± 4.1% vs. 34.2 ± 5.3%, overall *P* = 0.034, **Figure 1F**). Pairwise comparison showed that the difference was only statistically significant in the haplo-cord with HLA-A allele mismatch group vs. the haplo-cord with HLA-A allele match group (*P* = 0.013, **Figure 1F**). There was no significant difference in the cumulative incidence of grade II–IV aGVHD between the haplo-cord with HLA-A allele mismatch, haplo-HSCT, and haplo-cord with HLA-A allele match groups (46.7 ± 9.1% vs.34.2 ± 5.3% vs. 28.9 ± 3.9%, overall *P* = 0.068, respectively **Figure 1G**). The cumulative incidence of III–IV aGVHD was higher in the haplo-cord with HLA-A allele mismatch group than that in the haplo-HSCT or haplo-cord with HLA-A allele match groups (26.7 ± 8.1% vs. 14.1 ± 3.9% vs. 10.2 ± 2.6%, respectively, overall *P* = 0.038, **Figure 1H**). Pairwise comparison showed that the difference in grade III–IV aGVHD was only statistically significant in the haplo-cord with HLA-A allele mismatch group vs. the haplo-cord with HLA-A allele match pair group (*P* = 0.011, **Figure 1H**). There was no difference in the five-year cumulative incidence of overall cGVHD and moderate-to-severe cGVHD between the three subgroups (overall *P* = 0.236, **Figure 1I**; overall *P* = 0.513, **Figure 1J**).

There was also no difference in I–IV, II–IV, and III–IV aGVHD and total and moderate-to-severe cGVHD between the haplo-cord-HSCT subgroups grouped by matching or non-matching of the HLA-A antigen, HLA-B antigen and allele, and HLA-DRB1 antigen and allele (*P* > 0.05, **Supplemental Tables 1**, **2**) and between the haplo-cord-HSCT subgroups according to the counts of TNCs and CD34 ^+^ cells (greater or less than the median). In addition, no differences in the five types of GVHD mentioned above were observed among the haplo-cord-HSCT subgroups according to the degree of UCB and recipient HLA-matching (4/6, 5/6, or 6/6) (*P* > 0.05, **Supplemental Tables 1**, **2**).

In the entire cohort, the use of low-dose PTCy was a significantly protective factor for grade I–IV aGVHD and total cGVHD. the choice of haplo-cord-HSCT was another protective factor for total cGVHD in multivariate analysis (**Table 2**). Multivariate analysis showed that the matching of the HLA-A allele between the UCB and the recipient pair was only associated with lower I–IV and III–IV aGVHD in the haplo-cord-HSCT group (both *P* = 0.013) (**Table 3**).

**Table 2:** Multivariate analysis for GVHD and survival in the whole cohort.

Outcomes	Hazard ratio (95% CI)	*P*
I–IV aGVHD		
Donor gender (male vs. female)	0.810 (0.520–1.261)	0.350
Donor age < 40 years (yes vs. no)	1.012 (0.996–1.027)	0.132
Time from diagnosis to HSCT < 12 months (yes vs. no)	0.579 (0.363–0.982)	**0.042**
Group (haplo-HSCT vs. haplo-cord HSCT)	1.139 (1.139–0.982)	0.554
II–IV aGVHD		
Donor gender (male vs. female)	1.057 (0.656–1.702)	0.821
Donor age < 40 years (yes vs. no)	1.005 (0.988–1.022)	0.563
Time from diagnosis to HSCT < 12 months (yes vs. no)	1.259 (0.777–2.039)	0.394
Group (haplo-HSCT vs. haplo-cord HSCT)	0.654 (0.379–1.129)	0.127
III–IV aGVHD		
Donor gender (male vs. female)	0.863 (0.406–1.834)	0.702
Donor age < 40 years (yes vs. no)	1.020 (0.993–1.048)	0.141
Time from diagnosis to HSCT < 12 months (yes vs. no)	0.556 (0.228–1.358)	0.198
Group (haplo-HSCT vs. haplo-cord HSCT)	1.196 (0.569–2.513)	0.637
Total cGVHD		
Donor gender (male vs. female)	1.531 (00915–2.564)	0.105
Donor age < 40 years (yes vs. no)	1.016 (0.996–1.036)	0.116
Time from diagnosis to HSCT < 12 months (yes vs. no)	0.504 (0.260–0.974)	**0.042**
Group (haplo-HSCT vs. haplo-cord HSCT)	1.839 (1.079–3.130)	**0.025**
Moderate to severe cGVHD		
Donor gender (male vs. female)	2.329 (0.974–5.572)	0.057
Donor age < 40 years (yes vs. no)	1.023 (0.988–1.060)	0.196
Time from diagnosis to HSCT < 12 months (yes vs. no)	0.492 (0.142–1.709)	0.264
Group (haplo-HSCT vs. haplo-cord HSCT)	0.987 (0.348–2.800)	0.980
**OS**		
Donor gender (male vs. female)	1.428 (0.779–2.551)	0.229
Donor age < 40 years (yes vs. no)	1.016 (0.994–1.039)	0.164
Time from diagnosis to HSCT < 12 months (yes vs. no)	1.102 (0.597–2.035)	0.756
Group (haplo-HSCT vs. haplo-cord HSCT)	2.060 (1.162–3.654)	**0.013**
**GFFS**		
Donor gender (male vs. female)	1.268 (0.792–2.029)	0.323
Donor age < 40 years (yes vs. no)	1.014 (0.996–1.033)	0.131
Time from diagnosis to HSCT < 12 months (yes vs. no)	0.841 (0.490–1.445)	0.531
Group (haplo-HSCT vs. haplo-cord HSCT)	1.394 (0.861–2.255)	0.176

CI, confidence interva; vs, versus; Haplo-HSCT, haploidentical hematopoietic stem cell transplantation; Haplo-cord-HSCT, haploidentical hematopoietic stem cell transplantation with unrelated cord blood infusion. The bold values means P with statistical significance.

**Table 3:** Multivariate analysis for GVHD and survival in the haplo-cord-HSCT group.

Outcome	Hazard ratio (95% CI)	*P*
I–IV aGVHD		
Gender (male vs. female)	1.216 (0.736–2.009)	0.445
HLA-A allele match (yes vs. no)	0.472 (0.261–0.853)	**0.013**
TNCs < 1.80 × 10^7^/kg (yes vs. no)	1.464 (0.859–2.495)	0.162
CD34^+^ cells < 0.48 × 10^5^/kg (yes vs. no)	0.578 (0.333–1.004)	0.052
II–IV aGVHD		
Gender (male vs. female)	1.124 (0.627–2.016)	0.694
HLA-A allele match (yes vs. no)	0.548 (0.263–1.140)	0.107
TNCs < 1.80 × 10^7^/kg (yes vs. no)	0.562 (0.295–1.069)	0.079
CD34^+^ cells < 0.48 × 10^5^/kg (yes vs. no)	0.817 (0.438–1.524)	0.525
III–IV aGVHD		
Gender (male vs. female)	0.853 (0.355–2.053)	0.723
HLA-A allele match (yes vs. no)	0.319 (0.129–0.788)	**0.013**
TNCs < 1.80 × 10^7^/kg (yes vs. no)	0.866 (0.349–2.148)	0.756
CD34^+^ cells < 0.48 × 10^5^/kg (yes vs. no)	0.937 (0.381–2.305)	0.887
Total cGVHD		
Gender (male vs. female)	1.327 (0.700–2.516)	0.386
HLA-A allele match (yes vs. no)	0.700 (0.311–1.578)	0.390
TNCs < 1.80 × 10^7^/kg (yes vs. no)	0.616 (0.310–1.224)	0.167
CD34^+^ cells < 0.48 × 10^5^/kg (yes vs. no)	0.917 (0.472–1.781)	0.798
Moderate to severe cGVHD		
Gender (male vs. female)	1.199 (0.440–3.263)	0.723
HLA-A allele match (yes vs. no)	0.566 (0.171–1.878)	0.352
TNCs < 1.80 × 10^7^/kg (yes vs. no)	0.658 (0.221–1.957)	0.451
CD34^+^ cells < 0.48 × 10^5^/kg (yes vs. no)	1.272 (0.448–3.606)	0.651
OS		
Gender (male vs. female)	1.107 (0.509–2.404)	0.798
HLA-A allele match (yes vs. no)	0.572 (0.233–1.403)	0.222
TNCs < 1.80 × 10^7^/kg (yes vs. no)	0.943 (0.419–2.122)	0.887
CD34^+^ cells < 0.48 × 10^5^/kg (yes vs. no)	0.751 (0.332–1.695)	0.490
GFFS		
Gender (male vs. female)	0.937 (0.513–0.710)	0.831
HLA-A allele match (yes vs. no)	0.420 (0.220–0.801)	**0.008**
TNCs < 1.80 × 10^7^/kg (yes vs. no)	0.724 (0.381–1.378)	0.325
CD34^+^ cells < 0.48 × 10^5^/kg (yes vs. no)	1.013 (0.546–1.879)	0.969

CI, confidence interva; vs, versus; Haplo-cord HSCT, haploidentical hematopoietic stem cell transplantation with unrelated cord blood infusion; HLA, human leukocyte antigen; TNCs, total nucleated cells; ANC, absolute neutrophil count; aGVHD, acute graft-versus-host-disease; cGVHD, chronic GVHD. OS, overall syrvival; GFFS, GVHD-free/failure-free survival. Bold values means P with statistical significance.

### Infection

The incidence rate of bacterial and fungal infections was 64.3% in the haplo-HSCT group and 59.1% in the haplo-cord-HSCT (*P* = 0.422) (**Table 1**). The incidence of cytomegalovirus (CMV) viremia in the haplo-HSCT group was not different between the two groups (*P* = 0.779) (**Table 1**). Of the 84 patients in the haplo-HSCT group, 14 (16.7%) experienced EBV reactivation, while of the 171 patients in the haplo-cord-HSCT group, 24 (14.9%) experienced EBV viremia. After treatment with rituximab, the majority of patients with EBV viremia had a full recovery. However, one patient in the haplo-HSCT group finally died of EBV-associated post-transplant lymphoproliferative disease at day +395 (**Table 1**).

### TRM and Relapse

The median follow-up time in the living patients was 62 months (range, 11–156) in the haplo-HSCT group and 56 months (range, 12–117) in the haplo-cord-HSCT group (*P* = 0.407). No case had a recurrence of SAA during the follow-up period. The rate of TRM was higher in the haplo-HSCT group than in the haplo-cord-HSCT group (27.4% vs. 16.4%, *P* = 0.039). Further analysis showed that the prevalence of TRM caused by infection was higher in the haplo-HSCT group than that in the haplo-cord-HSCT group (13.7% vs. 4.1%, *P* = 0.016) (**Table 1**).

### Survival

**Figure 2:**
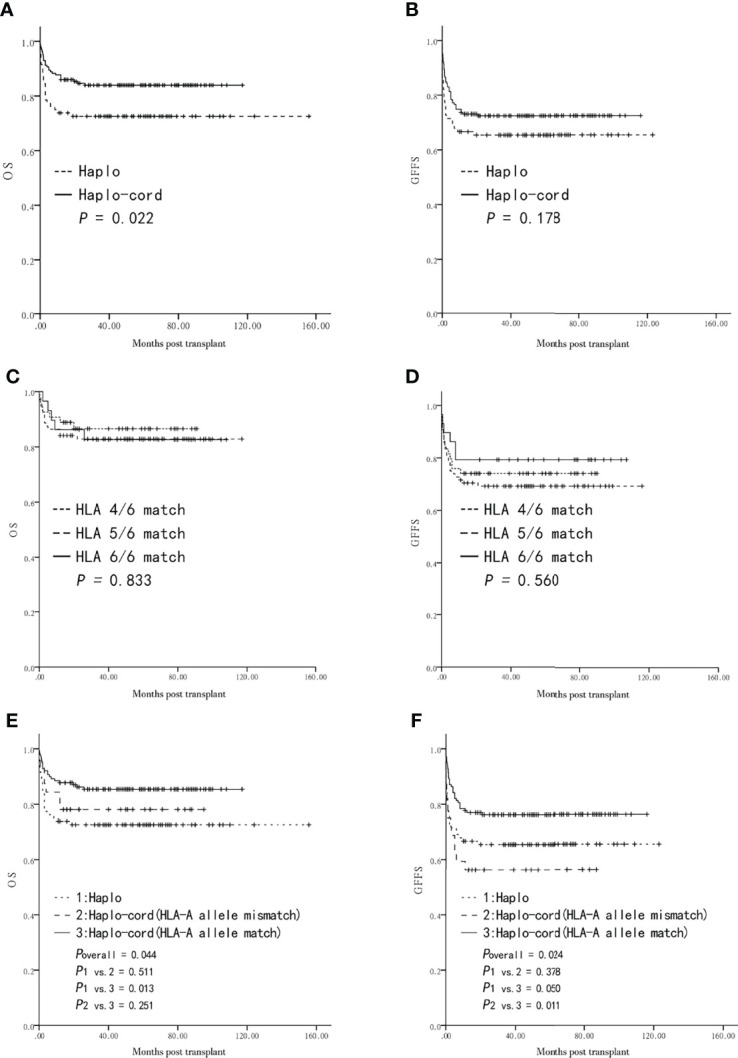
Overall survival (OS) and GVHD-free and failure-free survival (GFFS) after transplantation from Haplo-HSCT or Haplo-cord-HSCT (including subgroups). **(A)** OS between the two groups. **(B)** GFFS between the two groups. **(C)** OS among HLA 4, 5, 6 Haplo-cord-HSCT subgroups. **(D)** GFFS among HLA 4, 5, 6 Haplo-cord-HSCT subgroups. **(E)** OS among Haplo-HSCT group and Haplo-cord with a HLA-A match or mismatch subgroups. **(F)** GFFS among Haplo-HSCT group and Haplo-cord with a HLA-A match or mismatch subgroups.

The five-year probability of OS in the haplo-cord-HSCT group was higher than that in the haplo-HSCT group (84.0 ± 2.8% vs. 72.6 ± 4.9%, *P* = 0.022, **Figure 2A**). However, the five-year GFFS was not significantly different between the two groups (72.4 ± 3.4% vs. 65.4 ± 5.2%, *P* = 0.178, **Figure 2B**). Multivariate analysis showed that the choice of a haplo-cord-HSCT was associated with a longer OS (*P* = 0.013), whereas no beneficial factor for GFFS was identified (*P* > 0.05) (**Table 2**).

The UCB-related variables were then subjected to the univariate and multivariate analyses of the survival haplo-cord-HSCT group. The five-year probabilities of OS and GFFS were similar in the haplo-cord-HSCT subgroups according to the degree of HLA-matching (4/6, 5/6, 6/6) (86.5 ± 4.8% vs. 82.9 ± 4.0% vs. 82.6 ± 7.1%, *P* = 0.833, **Figure 2C**; 74.1 ± 6.0% vs. 69.2 ± 4.9% vs. 79.3 ± 7.5%, *P* = 0.560, **Figure 2D**).

Furthermore, no differences in the five-year OS and GFFS were observed between the matched and mismatched groups for the HLA-A antigen, HLA-B antigen, HLA-B allele, HLA-DRB1 antigen, and HLA-DRB1 allele (*P* > 0.05) (**Table 4**). Similarly, the OS and GFFS were not affected by the count of TNCs or CD34^+^ cells, sex (male or female), or blood type (match or mismatch) (*P* > 0.05) (**Table 4**).

**Table 4:** Univariate analysis of UCB-related characteristics on OS and GFFS.

Outcomes Characteristics	OS		GFFS	
	5-year estimated probability	*P*	5-year estimated probability	*P*
Sex				
Male	86.4 ± 3.5%	0.756	72.6 ± 4.3%	0.989
Female	83.1 ± 4.7%		72.3 ± 5.6%	
Blood type match				
Yes	82.4 ± 3.5%	0.322	78.6 ± 4.3%	0.151
No	87.4 ± 4.8%		65.3 ± 5.3%	
TNCs, × 10^7^/kg				
< 1.8	84.8 ± 4.0%	0.824	72.2 ± 5.0%	0.903
≥ 1.8	83.1 ± 4.0%		72.6 ± 4.7%	
CD34+ cells, × 10^5^/kg				
< 0.5	82.6 ± 4.0%	0.573	73.1 ± 4.6%	0.878
≥ 0.5	85.4 ± 4.1%		71.6 ± 5.1%	
HLA-A antigen match				
Yes	83.4 ± 3.1%	0.693	83.3 ± 7.6%	0.251
No	87.5 ± 6.8%		70.7 ± 3.8%	
HLA-A allele match				
Yes	85.4 ± 3.0%	0.251	76.2 ± 3.6%	**0.011**
No	78.1 ± 7.3%		56.3 ± 8.8%	
HLA-B antigen match				
Yes	83.7 ± 3.3 %	0.862	71.5 ± 4.0%	0.714
No	84.9 ± 5.7 %		75.6 ± 6.7%	
HLA-B allele match				
Yes	82.7 ± 3.5 %	0.474	69.8 ± 4.3 %	0.209
No	86.6 ± 4.8%		77.7 ± 5.7%	
HLA-DRB1 antigen match				
Yes	83.7 ± 3.2%	0.873	72.9 ± 3.8 %	0.685
No	84.7 ± 6.3 %		70.6 ± 7.8%	
HLA-DRB1 allele match				
Yes	83.7 ± 3.3%	0.962	71.9 ± 4.0 %	0.805
No	84.8 ± 5.3 %		73.9 ± 6.5%	

HLA, Human leukocyte antigen; UCB, Umbilical cord blood; OS, overall survival; GFFS, GVHD-free/failure-free survival; SE, Standard error; TNCs, total nucleated cells. Bold values means P with statistical significance.

Further survival comparison was performed for the haplo-cord with an HLA-A allele match, the haplo-cord with an HLA-A allele mismatch, and the haplo-HSCT groups. The five-year probabilities of OS and GFFS were higher in the former than in the latter two subgroups (85.4 ± 3.0% vs. 78.1 ± 7.3% vs. 72.6 ± 4.9%, overall *P* = 0.044, **Figure 2E**; 76.2 ± 3.6% vs. 56.3 ± 8.8% vs. 65.4 ± 5.2%, overall *P* = 0.024, **Figure 2F**). Pairwise comparison showed that OS was significantly different only in the haplo-cord with HLA-A allele match vs. the haplo-HSCT pairing (*P* = 0.013, **Figure 2E**), while GFFS was significantly different only in the haplo-cord with HLA-A allele match vs. the haplo-cord with HLA-A allele mismatch pairing (*P* = 0.011, **Figure 2F**).

In the multivariate analysis, a match between the UCB and the recipient pair for the HLA-A allele was only associated with a better GFFS in the haplo-cord-HSCT group (*P* = 0.013, **Table 4**).

## Discussion

Several single-arm studies have demonstrated the feasibility and effectiveness of haplo-cord-HSCT in SAA and other non-neoplastic and malignant hematologic disorders (13–15, 17). However, this approach for SAA is currently considered investigational and requires further study before firm recommendations can be made. In this double-arm multicenter study, we not only confirmed that OS is significantly increased in SAA patients receiving a haplo-cord-HSCT rather than a haplo-HSCT but also investigated the impact of UCB-related characteristics on outcomes using explorative subgroup analysis.

During the process of engraftment, we observed a similar median time of neutrophil and platelet engraftment between the haplo-HSCT and the haplo-cord-HSCT groups. Primary and secondary GF were also similar and low in both groups. The low incidence of GF can be explained by the following factors. First, because DSA is an important factor for GF in haplo-HSCT (23, 24), we attempted to avoid donors targeted by DSA or alternatively decreased the positive degree of DSA prior to transplantation. Second, all patients received the myeloablative conditioning regimen. Third, we ensured that repeated blood transfusions were minimized and also shortened the interval from diagnosis to transplantation. It is noteworthy that chimerism in the haplo-cord HSCT group all involved related haplografts without the evidence of UCB or mixed engraftment. This may be related to the following factors. First, because the majority of patients in our study were adults, the count of CD34^+^ cells was <0.5 × 10^5^ cells of the recipient’s weight, which was less than the CD34^+^ cell dose criteria in a single UCB unit (25). Second, in our protocol, the count of CD34^+^ cells relative to the recipient’s weight was more than 1 log lower in the UCB graft than in the haploidentical graft. Third, the unmanipulated haploidentical graft and conditioning regime with ATG were also different from the previous haplo-cord-HSCT protocol designed by foreign research teams (26–28). In patients with a successful engraftment, the incidence of aGVHD and moderate-to-severe cGVHD was similar between the haplo-HSCT and haplo-cord-HSCT groups, while the overall cGVHD in the haplo-cord-HSCT group tended to be lower than that observed in the haplo-HSCT group. These findings are consistent with those reported by an earlier study (15). This may be because UCB contains MSCs and CD4^+^CD25^+^ Tregs (29, 30), which have an immune regulatory role on the hematopoietic microenvironment and prevention of GVHD.

Although the five-year GFFS and relapse of the patients in the haplo-cord-HSCT and the haplo-HSCT groups was not statistically different, we found a significant difference in five-year OS and TRM between the two groups, both favoring the haplo-cord-HSCT group. Multivariate analysis showed that the most significant factor affecting OS was the treatment group, rather than the age and sex of the donor and the time from diagnosis to HSCT. Notably, survival in the haplo-HSCT group was relatively low compared to that described in another recent report (nine-year OS of 85.4%) (31). This may be because the proportion of young patients (<18 years old) was up to 50% in that study, whereas it was only 38% (<20 years old) in our study. In addition, this may be related to their coming from different multicenters. Among the causes of TRM, infection-related mortality was lower in the haplo-cord-HSCT group than in the haplo-HSCT group. Previous studies explored the relation between infection and GVHD (32, 33). Nevertheless, the limited number of infectious deaths does not allow determining the association between infectious deaths and cGVHD and comparing it between the two groups in the present study. A large, population-based analysis including the control of confounding factors is worthy of further study.

Our previous study has demonstrated that no differences in OS and GFFS were found between SAA subgroups after haplo-cord HSCT with 5/10 or 6/10–9/10 HLA-matched HIDs (17). Based on this, the improved survival in haplo-cord-HSCT group may have been affected by other factors, especially the coinfusion of UCB, which has seldom been the focus of previous studies. We next examined whether some characteristics of UCB were associated with survival outcomes, such as the degree of HLA matching, the locus of HLA disparity, and the number of CD34^+^ cells and TNCs. The results of our univariate analysis showed that an HLA-A allele match between the UCB and the recipient was the only beneficial factor for GFFS. Multivariate analysis also determined that the HLA-A allele match was a beneficial factor for GFFS and grade I–IV and III–IV aGVHD. Therefore, sharing the same HLA-A allele as the UCB in a haplo-cord-HSCT for SAA improves the possibility of GFFS by decreasing the incidence of grade III–IV aGVHD. Similar findings have been reported in a previous study (34). Furthermore, a low incidence of GVHD is often associated with an improvement in the health-related quality of life in patients following an allo-HSCT (16, 35). Although previous studies have reported that the degree of HLA matching and the number of CD34^+^ cells were important factors for UCB transplantation in pediatric patients with hematological diseases (36), no differences in OS and GFFS were found in our study between the haplo-cord HSCT subgroups with high or low TNCs and CD34^+^ cells and among subgroups with 4/6, 5/6, and 6/6 matching. As mentioned above, these inconsistent outcomes may be due to differences in the conditioning regime, grafts, and diseases in the different studies.

To examine the impact of an HLA-A allele match or mismatch between the UCB and the recipient on outcomes within and outside the haplo-cord group, we then performed a comparison between these two corresponding groups and the haplo-HSCT group. On the one hand, the incidence of GVHD was lower in the haplo-cord with the HLA allele match group than that observed in the haplo-cord with HLA allele mismatch and haplo-HSCT groups. No significant difference was found between the latter two groups. On the other hand, the OS and GFFS rates were higher in the haplo-cord with the HLA allele match group than that in the haplo-cord with HLA allele mismatch and haplo-HSCT groups, with no significant difference between the latter two groups. These preliminary results suggest that when an SAA patient undergoes a haplo-HSCT, the combination of UCB with an HLA-A allele match with the recipient should be preferred, while the option with an HLA-A allele mismatch is still worth considering. A future prospective study in a larger number of patients is required to confirm these conclusions.

In conclusion, this multiple-centered cohort study in SAA patients had two major findings (1): preliminary data showed that a haplo-cord-HSCT had a better OS and lower TRM compared with that observed for a haplo-HSCT alone, and (2) on the basis of at least an HLA 4/6 match between the UCB and the recipient pair, UCB’ selection should primarily consider the degree of HLA-A allele match, rather than the count of TNCs/CD 34^+^ cells, sex, and blood type. These algorithms may be helpful for UCB selection in SAA patients undergoing a haplo-cord-HSCT.

## Data Availability Statement

The original contributions presented in the study are included in the article/**Supplementary Material**. Further inquiries can be directed to the corresponding authors.

## Ethics Statement

Written informed consent was obtained from the individual(s), and minor(s)’ legal guardian/next of kin, for the publication of any potentially identifiable images or data included in this article.

## Author Contributions

M-QL, Y-MZ, and W-JJ wrote the manuscript and performed the analysis. D-PW, MM, and L-ML designed the protocol. All authors contributed patients, provided clinical and laboratory data, and revised and corrected the manuscript.

## Conflict of Interest

The authors declare that the research was conducted in the absence of any commercial or financial relationships that could be construed as a potential conflict of interest.

## Publisher’s Note

All claims expressed in this article are solely those of the authors and do not necessarily represent those of their affiliated organizations, or those of the publisher, the editors and the reviewers. Any product that may be evaluated in this article, or claim that may be made by its manufacturer, is not guaranteed or endorsed by the publisher.
