# Supplementary material for: Comparison of Haploidentical Hematopoietic Stem Cell Transplant With or Without Unrelated Cord Blood Infusion in Severe Aplastic Anemia: Outcomes of a Multicenter Study
Source: Front Immunol. 2022 Jun 23;13:912917. doi: 10.3389/fimmu.2022.912917 (PMC9259833; doi:10.3389/fimmu.2022.912917)
Supplement: Supplementary file 1 [file Table_1.docx]

**Supplemental Table 1. Univariate analysis of UCB-related characteristics on the development of aGVHD**

| **Outcome**  **Characteristic** | **aGVHD** | | | | | |
| --- | --- | --- | --- | --- | --- | --- |
|  | I–IV | ***P*** | II–IV | P | III–IV | ***P*** |
| HLA-A antigen matched |  |  |  |  |  |  |
| Yes | 40.8 ± 4.1% | 0.123 | 34.5 ± 4.0% | 0.139 | 14.1 ± 2.9% | 0.504 |
| No | 21.7 ± 8.6% |  | 17.4 ± 7.9% |  | 8.7 ± 5.9% |  |
| HLA-B antigen matched |  |  |  |  |  |  |
| Yes | 39.4 ± 4.3 % | 0.568 | 33.1± 4.2% | 0.635 | 14.2 ± 3.1% | 0.536 |
| No | 34.2 ± 7.7 % |  | 28.9 ± 7.4% |  | 10.5 ± 5.0% |  |
| HLA-B allele matched |  |  |  |  |  |  |
| Yes | 40.9 ± 4.7 % | 0.362 | 34.5 ± 4.5 % | 0.386 | 13.6 ± 3.3 % | 0.886 |
| No | 32.7 ± 6.3% |  | 27.2 ± 6.0% |  | 12.7 ± 4.5% |  |
| HLA-DRB1 antigen matched |  |  |  |  |  |  |
| Yes | 37.6 ± 4.2% | 0.600 | 31.6 ± 4.0 % | 0.605 | 12.8 ± 2.9 % | 0.642 |
| No | 40.6 ± 8.7 % |  | 34.4 ± 8.4% |  | 15.6 ± 6.4% |  |
| HLA-DRB1 allele matched |  |  |  |  |  |  |
| Yes | 39.7 ± 4.4% | 0.583 | 32.2 ± 4.2 % | 0.973 | 14.0 ± 3.2 % | 0.691 |
| No | 34.1 ± 7.1 % |  | 31.8 ± 7.0% |  | 11.4 ± 4.8% |  |
| TNCs, × 10^7^/kg |  |  |  |  |  |  |
| < 1.8 | 32.9 ± 5.2% | 0.172 | 28.0 ± 5.0% | 0.274 | 13.4 ± 3.8% | 0.972 |
| ≥ 1.8 | 43.5 ± 5.4% |  | 36.1 ± 5.3% |  | 13.3 ± 3.7% |  |
| CD34+ cells, × 10^5^/kg |  |  |  |  |  |  |
| < 0.5 | 40.5 ± 5.4% | 0.574 | 33.3 ± 5.1% | 0.776 | 13.1 ± 3.7% | 0.894 |
| ≥ 0.5 | 35.8 ± 5.3% |  | 30.9 ± 5.1% |  | 13.6 ± 3.8% |  |
| Degree of HLA matched |  |  |  |  |  |  |
| 4/6 | 38.6 ± 7.3% | 0.855 | 31.8 ± 6.0% | 0.631 | 18.2 ± 5.8% | 0.439 |
| 5/6 | 38.6 ± 5.2% |  | 34.1 ± 5.1% |  | 12.5 ± 3.5% |  |
| 6/6 | 36.4 ± 8.4% |  | 27.3 ± 7.8% |  | 9.1 ± 5.0% |  |
