# Supplementary material for: Comparison of Haploidentical Hematopoietic Stem Cell Transplant With or Without Unrelated Cord Blood Infusion in Severe Aplastic Anemia: Outcomes of a Multicenter Study
Source: Front Immunol. 2022 Jun 23;13:912917. doi: 10.3389/fimmu.2022.912917 (PMC9259833; doi:10.3389/fimmu.2022.912917)
Supplement: Supplementary file 2 [file Table_2.docx]

**Supplemental Table 2. Univariate analysis of UCB related characteristics on the development of cGVHD**

| **Outcomes**  **Characteristics** | **cGVHD** | | | |
| --- | --- | --- | --- | --- |
|  | **Total** | ***P*** | **Moderate to severe** | ***P*** |
| HLA-A antigen matched |  |  |  |  |
| Yes | 20.6 ± 4.0% | 0.091 | 12.3 ± 3.0% | 0.185 |
| No | 9.8 ± 6.6% |  | 3.5 ± 3.4% |  |
| HLA-B antigen matched |  |  |  |  |
| Yes | 26.2 ± 4.1 % | 0.663 | 11.4 ± 3.0% | 0.685 |
| No | 29.9 ± 8.1 % |  | 8.1 ± 4.5% |  |
| HLA-B allele matched |  |  |  |  |
| Yes | 30.9 ± 4.6 % | 0.095 | 11.9 ± 3.2 % | 0.432 |
| No | 19.5 ± 5.9% |  | 9.5 ± 4.1% |  |
| HLA-DRB1 antigen matched |  |  |  |  |
| Yes | 30.2 ± 4.2% | 0.101 | 10.0 ± 2.7 % | 0.542 |
| No | 13.7 ± 6.3 % |  | 13.7 ± 6.4% |  |
| HLA-DRB1 allele matched |  |  |  |  |
| Yes | 26.6 ± 4.3% | 0.828 | 8.2 ± 2. % | 0.099 |
| No | 28.0 ± 7.2 % |  | 17.5 ± 6.0% |  |
| TNCs, × 10^7^/kg |  |  |  |  |
| < 1.8 | 31.0 ± 5.4% | 0.267 | 10.9 ± 3.6% | 0.992 |
| ≥ 1.8 | 23.0 ± 4.9% |  | 10.3 ± 3.5% |  |
| CD34+ cells, × 10^5^/kg |  |  |  |  |
| < 0.5 | 24.3 ± 5.0% | 0.511 | 10.7 ± 3.6% | 0.973 |
| ≥ 0.5 | 19.7 ± 5.4% |  | 10.6 ± 3.5% |  |
| Degree of HLA-matched |  |  |  |  |
| 4/6 | 24.7 ± 6.8% | 0.932 | 17.2 ± 6.0% | 0.286 |
| 5/6 | 28.2 ± 5.1% |  | 8.8 ± 3.2% |  |
| 6/6 | 27.0 ± 8.2% |  | 6.7 ± 4.6% |  |
