# Supplementary material for: Comparison of Haploidentical Hematopoietic Stem Cell Transplant With or Without Unrelated Cord Blood Infusion in Severe Aplastic Anemia: Outcomes of a Multicenter Study
Source: Front Immunol. 2022 Jun 23;13:912917. doi: 10.3389/fimmu.2022.912917 (PMC9259833; doi:10.3389/fimmu.2022.912917)
Supplement: Supplementary file 3 [file Table_3.docx]

**Supplemental Table 3. patient number in the two groups from different centers**

| **Centers** | **Haplo-HSCT**  **(n = 84)** | **Haplo-cord-HSCT**  **(n = 171)** |
| --- | --- | --- |
| The First Affiliated Hospital of Soochow University, Jiangsu Institute of Hematology, Key Laboratory of Thrombosis and Hemostasis of Ministry of Health, Collaborative Innovation Center of Hematology | 58 | 149 |
| Haikou Municipal People’s Hospital, Affiliated Haikou Hospital of Xiangya Medical College, Central South University | 2 | 0 |
| The Affiliated Huai’an Hospital of Xuzhou Medical University and the Second People’s Hospital of Huai’an, Huai’an | 3 | 2 |
| Xian Yang Central Hospital, Xianyang | 2 | 0 |
| Soochow Hopes Hematonosis Hospital | 19 | 20 |
